# sVEGFR1 Is Enriched in Hepatic Vein Blood—Evidence for a Provisional Hepatic Factor Candidate?

**DOI:** 10.3389/fped.2021.679572

**Published:** 2021-06-14

**Authors:** Andrew D. Spearman, Ankan Gupta, Amy Y. Pan, Todd M. Gudausky, Susan R. Foerster, G. Ganesh Konduri, Ramani Ramchandran

**Affiliations:** ^1^Division of Cardiology, Department of Pediatrics, Medical College of Wisconsin, Children's Wisconsin, Herma Heart Institute, Milwaukee, WI, United States; ^2^Cardiovascular Center, Medical College of Wisconsin, Milwaukee, WI, United States; ^3^Division of Neonatology, Department of Pediatrics, Medical College of Wisconsin, Children's Wisconsin, Milwaukee, WI, United States; ^4^Division of Quantitative Health Sciences, Department of Pediatrics, Medical College of Wisconsin, Children's Wisconsin, Milwaukee, WI, United States

**Keywords:** congenital heart disease, single ventricle, vascular remodeling, pulmonary arteriovenous malformation (AVM), Glenn, vascular endothelial growth factor

## Abstract

**Background:** Pulmonary arteriovenous malformations (PAVMs) are common sequelae of palliated univentricular congenital heart disease, yet their pathogenesis remain poorly defined. In this preliminary study, we used paired patient blood samples to identify potential hepatic factor candidates enriched in hepatic vein blood.

**Methods:** Paired venous blood samples were collected from the hepatic vein (HV) and superior vena cava (SVC) from children 0 to 10 years with univentricular and biventricular congenital heart disease (*n* = 40). We used three independent protein analyses to identify proteomic differences between HV and SVC blood. Subsequently, we investigated the relevance of our quantified protein differences with human lung microvascular endothelial assays.

**Results:** Two independent protein arrays (semi-quantitative immunoblot and quantitative array) identified that soluble vascular endothelial growth factor receptor 1 (sVEGFR1) is significantly elevated in HV serum compared to SVC serum. Using ELISA, we confirmed the previous findings that sVEGFR1 is enriched in HV serum (*n* = 24, *p* < 0.0001). Finally, we studied the quantified HV and SVC serum levels of sVEGFR1 *in vitro*. HV levels of sVEGFR1 decreased tip cell selection (*p* = 0.0482) and tube formation (fewer tubes [*p* = 0.0246], shorter tube length [*p* = 0.0300]) *in vitro* compared to SVC levels of sVEGFR1.

**Conclusions:** Based on a small heterogenous cohort, sVEGFR1 is elevated in HV serum compared to paired SVC samples, and the mean sVEGFR1 concentrations in these two systemic veins cause pulmonary endothelial phenotypic differences *in vitro*. Further research is needed to determine whether sVEGFR1 has a direct role in pulmonary microvascular remodeling and PAVMs in patients with palliated univentricular congenital heart disease.

## Introduction

Pulmonary arteriovenous malformations (PAVMs) are common sequelae of surgical palliation for univentricular congenital heart disease (CHD) (1, 2). Clinical observations indicate that an unidentified factor in hepatic vein (HV) blood, called hepatic factor, is protective against development of PAVMs and can resolve existing PAVMs (3). HV blood, and thus hepatic factor, is excluded from pulmonary blood flow after palliation with a superior cavopulmonary connection (or Glenn). PAVMs develop after superior cavopulmonary palliation when pulmonary blood flow is provided exclusively from superior vena cava (SVC) blood. After subsequent palliation with a total cavopulmonary connection (or Fontan), HV blood is re-directed to the pulmonary vasculature and PAVMs usually resolve. The hepatic factor hypothesis was proposed nearly 30 years ago, yet hepatic factor is still not identified and the pathophysiology of CHD-associated PAVMs remains incompletely understood (4).

To better understand the pathophysiology of CHD-associated PAVMs, we began collecting paired patient blood samples from the HV and SVC. Using these paired samples, we previously published a preliminary report that HV and SVC serum differentially impact lung microvascular endothelial cells *in vitro* (5). Because previous studies using paired venous blood samples have so far failed to identify hepatic factor candidates (6, 7), we then sought to identify hepatic factor candidates by screening HV and SVC blood. Under the premise that the unidentified hepatic factor is present in relatively high concentrations in HV blood but negligible levels in SVC blood, we screened HV and SVC blood in both a selective and unbiased manner. Secondarily, we used *in vitro* endothelial assays to identify whether quantified protein differences in our candidate factor (soluble vascular endothelial growth factor receptor 1 [sVEGFR1]) cause distinct endothelial phenotypes.

## Methods

### Blood Collection

Blood samples were collected from children 0 to 10 years old undergoing cardiac catheterization at Children's Wisconsin after obtaining parental written informed consent, as previously described (5). Samples were collected from two anatomic locations: (#1) SVC, and (#2) immediately cephalad to HV insertion into the inferior vena cava (IVC). Site #2 will be referred to as HV. Blood samples were collected under the premise that the unidentified hepatic factor is enriched in HV blood (from patients with biventricular and univentricular anatomy) and at negligible levels in SVC blood. After collection, serum was isolated, aliquoted, and stored at −80°C. Due to limited blood sample volumes, not all serum protein assays were performed for all patients in this study. This study was approved by the local Institutional Review Board.

Patient variables were collected from the electronic medical record. Assessment of PAVMs was based on clinically available data. PAVMs were diagnosed using bubble contrast echocardiography during cardiac catheterization. For patients without a bubble contrast echocardiogram, PAVMs were suspected in all patients with univentricular CHD and Glenn circulation because previous data indicate that PAVMs universally develop after Glenn palliation (2). PAVMs were also suspected in patients with non-fenestrated Fontan circulation with peripheral oxygen saturations <90% and no significant veno-venous collaterals.

### Semi-Quantitative Serum Protein Array

We custom designed a membrane-based antibody array (immunoblot) to semi-quantitatively assess serum levels of proteins involved in microvascular remodeling (RayBiotech, AAH-cust-m). Samples were diluted 2-fold and assayed in duplicate, per manufacturer recommendations.

### Quantitative Serum Protein Array

Concurrently with our custom immunoblot array, we sought to directly quantify a larger unbiased array of serum proteins through a commercial proteomic service (RayBiotech, Quantibody array, QAH-CAA-440-1) in a separate cohort of patient samples. Patient serum samples were sent to the commercial proteomic service, verified by quality control testing, and 440 target proteins were quantified in quadruplicate.

### Quantitative sVEGFR1 ELISA

Because sVEGFR1 was enriched in HV serum in two previous assays, we used a commercially available VEGFR1 sandwich ELISA (Abcam, ab195210) to quantify serum VEGFR1 levels in an independent cohort of patient samples. Samples were diluted 2-fold and assayed in duplicate, per manufacturer recommendations.

### Cell Culture

Primary human pulmonary microvascular endothelial cells (HPMECs) isolated from the lung of a single donor were purchased and used for all experiments (Promocell, #C-12281). Mycoplasma-free cells were grown and maintained in Endothelial cell growth medium MV (Promocell, #C-22020) and studied at passage 4–6.

### *In vitro* Tip Cell Selection: CD34 Quantification

Contact inhibited HPMECs were seeded in 6-cm dishes (0.4 × 10^6^ cells/dish) in complete media and supplemented with recombinant human VEGF-A (ThermoFisher, #PHC9394) and/or recombinant human sVEGFR1 (R&D Systems, #321-FL) for 24 h. Treated cells were then trypsinized with TrypLE express (ThermoFisher, #12604013), washed in FACS buffer (PBS, 10% fetal bovine serum [FBS]), and surface stained with the following cocktail of conjugated antibodies: (CD34-PE [BD-Biosciences, #560941], CD31-APC/Cy7 [Biolegend, #303120], VE-cadherin-FITC [BD-Biosciences, #560874], VEGFR2-PerCP/Cy5.5 [Biolegend, #359908], and Live/Dead yellow stain [ThermoFisher, #L34968]; all diluted 1:200). Samples were acquired on a BD Fortessa flow cytometer and data were analyzed with FlowJo software (version 10.5.3). Only viable cells (negative for Live/Dead yellow stain) that were positive for both endothelial markers (CD31 and VE-cadherin) were used for analysis.

### *In vitro* Angiogenesis: Tube Formation

One day prior to tube formation assay, reduced growth factor Matrigel (VWR, #47743-718) was thawed overnight at 4°C and contact inhibited HPMECs were serum-starved overnight in 0.5% FBS. Undiluted Matrigel was added (65 μL) to each well of a 96 well-plate and allowed to solidify at 37°C for 1 h. HPMECs were then trypsinized, counted, and 1 x 10^4^ cells/well were added in serum free media with VEGF-A and/or sVEGFR1 treatment (triplicate). HPMECs were then incubated at 37°C for 4 h. Tube formation was visualized using Keyence BZ-X700 bright-field/fluorescent microscope (Japan). Images were empirically obtained from the center of each well and analyzed with ImageJ using the “Angiogenesis Analyzer” function to quantify number of nodes, number of tubes, and tube length. Triplicated samples were averaged for each experiment, and the experiment was repeated four independent times for statistical analysis. Results are reported relative to control (HPMEC treated with serum free media alone).

### Statistical Analysis

Data are expressed as median (interquartile range [IQR]) or mean ± standard error of the mean (SEM). For custom immunoblot and ELISA, paired *t* test was performed for protein comparisons between HV and SVC. If normality assumption was not met, log2 transformation was employed or Wilcoxon Signed Rank test was used. For quantitative protein array, log2 fold change was calculated for each paired sample and statistical significance was accepted at false discovery rate (FDR) < 0.2 and average absolute log2 fold change > 2. For FACS and tube formation assays, one-way ANOVA with Tukey multiple comparison test was performed. Unless otherwise noted, statistical significance was accepted at *P* < 0.05. Analyses were performed using GraphPad Prism 8 (GraphPad Software, LLC), SAS 9.4 (SAS Institute Inc., Cary, NC), and R package gplots (8).

## Results

### Patient Characteristics

Blood samples from 40 patients at Children's Wisconsin were collected and used for all reported assays (Table 1), including patients with univentricular (18/40, 45.0%) and biventricular anatomy (22/40, 55.0%). Of those with univentricular CHD, 1 (5.6%) patient had Norwood/Sano circulation, 13 (72.2%) patients had superior cavopulmonary circulation, and 4 (22.2%) had Fontan circulation. PAVMs were diagnosed (*n* = 3) or suspected (*n* = 12) in 15 (37.5%) patients, though contrast studies were not systematically performed. Detailed patient characteristics for each individual assay can be found in Table 1. Serum samples used for all experiments were independent samples except for one male patient with double inlet left ventricle, superior cavopulmonary circulation, and suspected PAVMs who had samples used for both the semi-quantitative immunoblot and quantitative protein array.

**Table 1:** Patient demographics.

		**Overall Cohort**	**Assay #1: Semi-quantitative immunoblot**	**Assay #2: Quantitative protein array**	**Assay #3: ELISA**
N		40	10	7	24
Age (months)		31.5 [23.5, 57.0]	53.5 [31.0, 71.5]	47 [13, 54]	28.0 [17.5, 43.75]
Male		15 (37.5)	4 (40.0)	2 (28.6)	10 (41.7)
Univentricular CHD		18 (45.0)	4 (40.0)	4 (57.1)	11 (45.8)
PAVMs		15 (37.5)	3 (30.0)	4 (57.1)	9 (37.5)
Oxygen saturation (SpO_2_)		0.93 [0.86, 0.98]	0.95 [0.91, 0.99]	0.89 [0.83, 0.99]	0.92 [0.86, 0.98]
Primary Cardiac Diagnosis					
Biventricular	Atrial Septal Defect	4	2	2	–
	Congenitally corrected transposition	1	–	–	1
	Coarctation of aorta	1	–	1	–
	Patent ductus arteriosus	7	1	–	6
	Pulmonary hypertension	3	1	—	2
	Pulmonary valve stenosis	1	–	–	1
	Shone's complex	1	–	–	1
	Tetralogy of Fallot	4	2	–	2
Univentricular	Atrioventricular septal defect	3	–	–	3
	Double inlet left ventricle	1^#^	1	1	–
	Double outlet right ventricle	1	–	1	–
	Hypoplastic left heart syndrome	11	3	1	7
	Pulmonary atresia intact ventricular septum	1	–	–	1
	Tricuspid atresia	1	–	1	–

*Data are expressed as N (%) or median [IQR]. CHD, congenital heart disease, PAVMs, pulmonary arteriovenous malformations*.

# *Serum from a male patient with double inlet left ventricle and superior cavopulmonary circulation was used for both assay #1 and #2. All other samples for all assays were independent samples*.

### Serum Protein Array—Semi-Quantitative Immunoblot

**Figure 1:**
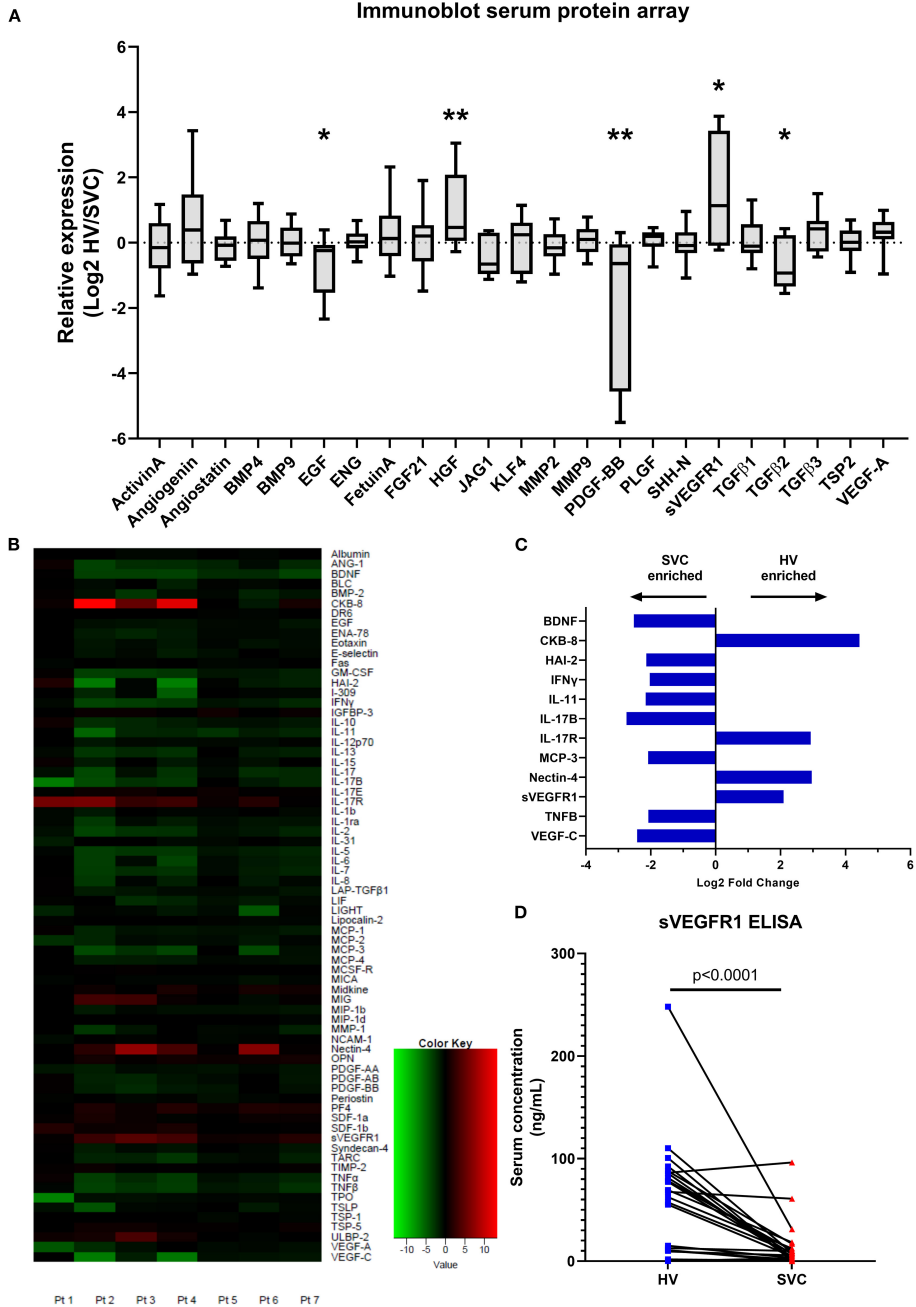
Serum protein arrays quantifying protein targets in paired patient samples from the hepatic vein (HV) and superior vena cava (SVC). **(A)** Immunoblot array quantifying serum protein levels of circulating proteins involved in microvascular remodeling. Protein concentrations in paired patient samples are expressed as relative expression (HV/SVC) after Log2 transformation. Boxes indicate median and interquartile range; whiskers indicate 95% confidence intervals; *n* = 10. * indicates *p* < 0.05, ** indicates *p* < 0.01. **(B)** Heatmap from the quantitative serum protein array (440 target proteins) showing raw log2 fold change of serum proteins with FDR <0.2; *n* = 7. Positive log2 fold change (red) indicates greater HV serum concentration. **(C)** Bar graph showing average log2 fold change of serum proteins with FDR <0.2 and absolute average log2 fold change >2. **(D)** Paired circulating sVEGFR1 levels quantified using sandwich ELISA (Abcam; *n* = 24).

To identify if various circulating proteins involved in vascular remodeling were present in different concentrations in HV and SVC serum, we generated a custom membrane-based antibody-array (immunoblot) to semi-quantitatively compare protein levels (Figure 1A). Using the custom immunoblot (*n* = 10), we observed that HV serum had significantly greater levels of sVEGFR1 (*p* = 0.0370) and hepatocyte growth factor (HGF; *p* = 0.0098) compared to SVC serum. Epidermal growth factor (EGF; *p* = 0.0440), platelet derived growth factor-BB (PDGF-BB; *p* = 0.0098), and transforming growth factor β2 (TGF-β2; *p* = 0.0230) were enriched in SVC serum. No other proteins had statistically significant differences.

### Serum Protein Array—Quantitative Array

To independently verify our semi-quantitative array and investigate protein levels in HV and SVC serum in an unbiased manner, we utilized commercial proteomic testing to quantify protein levels in paired samples from 7 patients. Of the 440 target proteins quantified, 72 proteins were preliminarily identified as having statistically significant differences in serum levels in HV vs. SVC (FDR <0.2) (Figure 1B, Supplementary Table 1). To identify protein differences between HV and SVC serum more rigorously, we identified proteins with FDR <0.2 and average absolute log2 fold change > 2 (Figure 1C). From this list of 12 proteins, 4 were enriched in HV serum (CKB-8, IL-17R, Nectin-4, and sVEGFR1), whereas the remainder were enriched in SVC serum.

### Serum sVEGFR1 Quantification

Because sVEGFR1, a circulating protein that functions to sequester VEGF-A, was enriched in HV serum in our two previous independent assays and increased VEGF signaling is associated with CHD-associated PAVMs (9), we sought to confirm that sVEGFR1 is enriched in HV serum. To do this, we quantified sVEGFR1 levels in HV and SVC serum in a larger cohort of samples with a commercially available sandwich ELISA (Figure 1D). Independent quantification of sVEGFR1 serum levels with ELISA again demonstrated significantly greater levels of sVEGFR1 in HV serum (HV 62.43 ± 10.97 ng/ml, SVC 12.93 ± 4.51 ng/ml; *p* < 0.0001; *n* = 24). After adjusting for patient variables (age, gender, univentricular/biventricular, and oxygen saturation [SpO_2_]), sVEGFR1 remained statistically increased in HV serum compared to SVC serum (Supplementary Table 2, Supplementary Figure 1).

### *In vitro* Tip Cell Selection: CD34 Quantification

**Figure 2:**
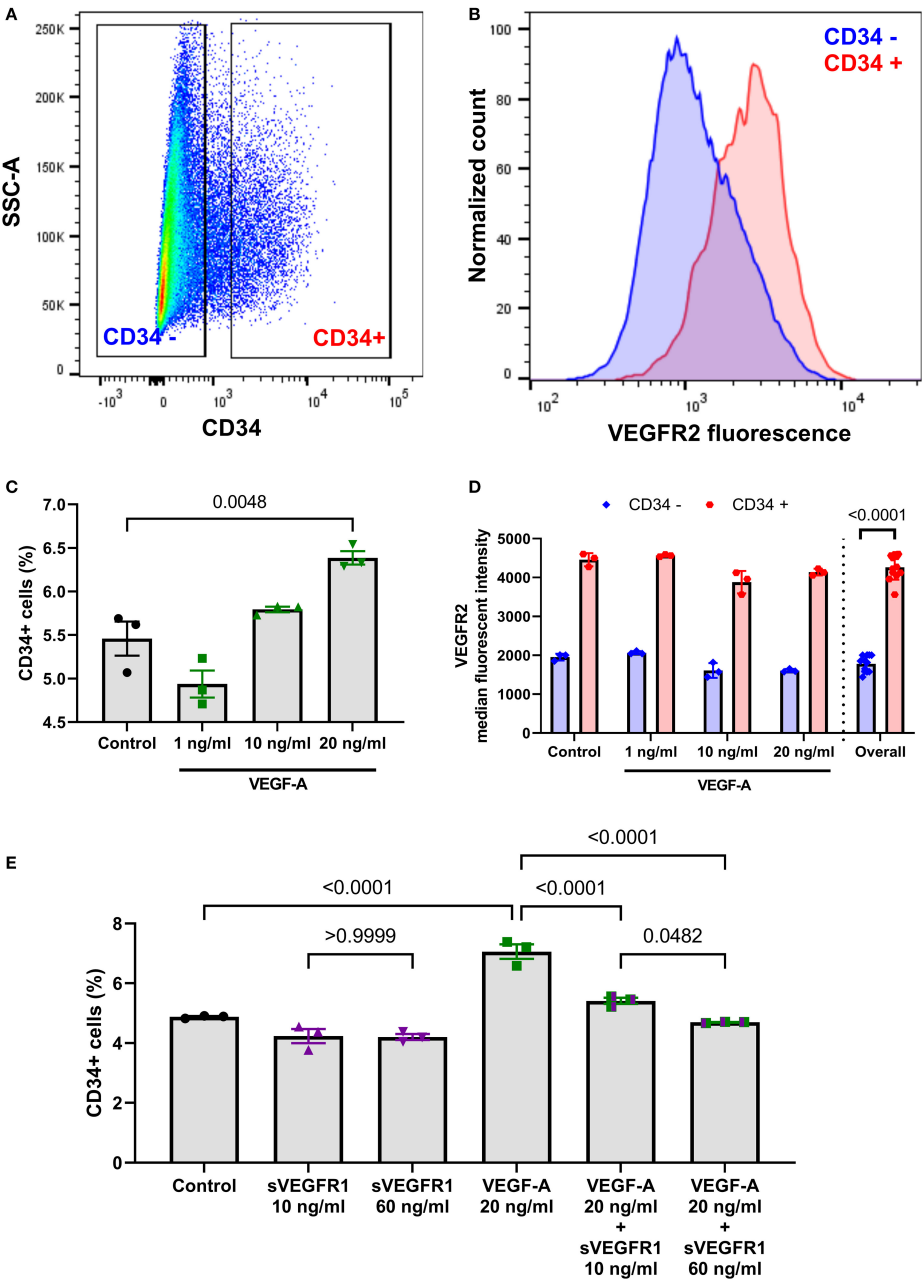
Fluorescence-activated cell sorting analysis of tip cell marker CD34. **(A)** Scatter plot of HPMECs showing a distribution of CD34+ and CD34– HPMECS with exclusion of intermediate HPMECs. **(B)** Histogram comparison of cell surface VEGFR2 fluorescence between CD34+ and CD34– HPMECs. **(C)** Scatter dot plot with bars quantifying CD34+ HPMECs after treatment with control or VEGF-A treatment showing a dose response of CD34+ cells to greater VEGF-A concentration (*p* = 0.0048; one-way ANOVA with Tukey multiple comparison test). **(D)** Quantification of cell surface VEGFR2 median fluorescent intensity between CD34+ and CD34– HPMECs for each individual VEGF-A treatment and overall combined (*p* < 0.0001, *t*-test). **(E)** Scatter dot plot with bars quantifying CD34+ HPMECs after treatment with control, sVEGFR1 alone (10 or 60 ng/ml), VEGF-A alone (20 ng/ml), or VEGF-A (20 ng/ml) + sVEGFR1 (10 or 60 ng/ml). VEGF-A stimulated HPMECs had significantly fewer CD34+ cells with HV-related sVEGFR1 treatment (60 ng/ml) compared to SVC-related sVEGFR1 treatment (10 ng/ml) (*p* = 0.0482; one-way ANOVA with Tukey multiple comparison test). Mean ± SEM. HPMECs- human pulmonary microvascular endothelial cells.

Based on this initial sVEGFR1 quantification, we then sought to determine if the mean concentrations of sVEGFR1 in HV serum (~60 ng/ml) and SVC serum (~10 ng/ml) could cause different pulmonary microvascular endothelial cell phenotypes *in vitro*. Soluble VEGFR1 is known to regulate microvascular angiogenesis by inhibiting tip cell selection (10, 11), which are the cells that form the leading edge of an angiogenic sprout. Numerous molecular markers are used to identify tip cells, including previous studies that identified CD34 as a reliable tip cell population marker *in vitro* (12–14). Thus, we sought to quantify CD34+ cells using HPMECS treated with VEGF-A and either HV or SVC-related sVEGFR1 concentrations. To do this, we first identified a CD34+ population of HPMECs *in vitro* that highly express VEGFR2 levels compared to CD34– HPMECs, which is consistent with a tip cell molecular phenotype (Figures 2A,B). Then, with increasing doses of VEGF-A treatment, we found that the CD34+ population proportionally increases with VEGF-A concentration, and CD34+ HPMECs are enriched in VEGFR2 expression (Figures 2C,D). These findings are consistent with previous studies and support that CD34+ is a tip cell population marker *in vitro* for HPMECs (12–14). Finally, we treated HPMECs with the combination of VEGF-A + sVEGFR1 (10 or 60 ng/ml). We observed that both concentrations of sVEGFR1 decreased the CD34+ population compared to VEGF-A alone (*p* < 0.0001 for both, Figure 2E); however, HV-related sVEGFR1 treatment (60 ng/ml) decreased the CD34+ population to a significantly greater degree than SVC-related sVEGFR1 treatment (10 ng/ml) (HV: 4.69 ± 0.01%, SVC: 5.41 ± 0.10%, *p* = 0.0482, Figure 2E). Treatment with sVEGFR1 alone (10 or 60 ng/ml) without simultaneous VEGF-A stimulation showed no difference between the different sVEGFR1 concentrations (HV: 4.20 ± 0.10%, SVC: 4.23 ± 0.24%, *p* > 0.9999, Figure 2E). These findings suggest that the different sVEGFR1 concentrations in HV and SVC serum differentially impact tip cell selection of VEGF-stimulated lung microvascular endothelial cells *in vitro*.

### *In vitro* Angiogenesis: Tube Formation

**Figure 3:**
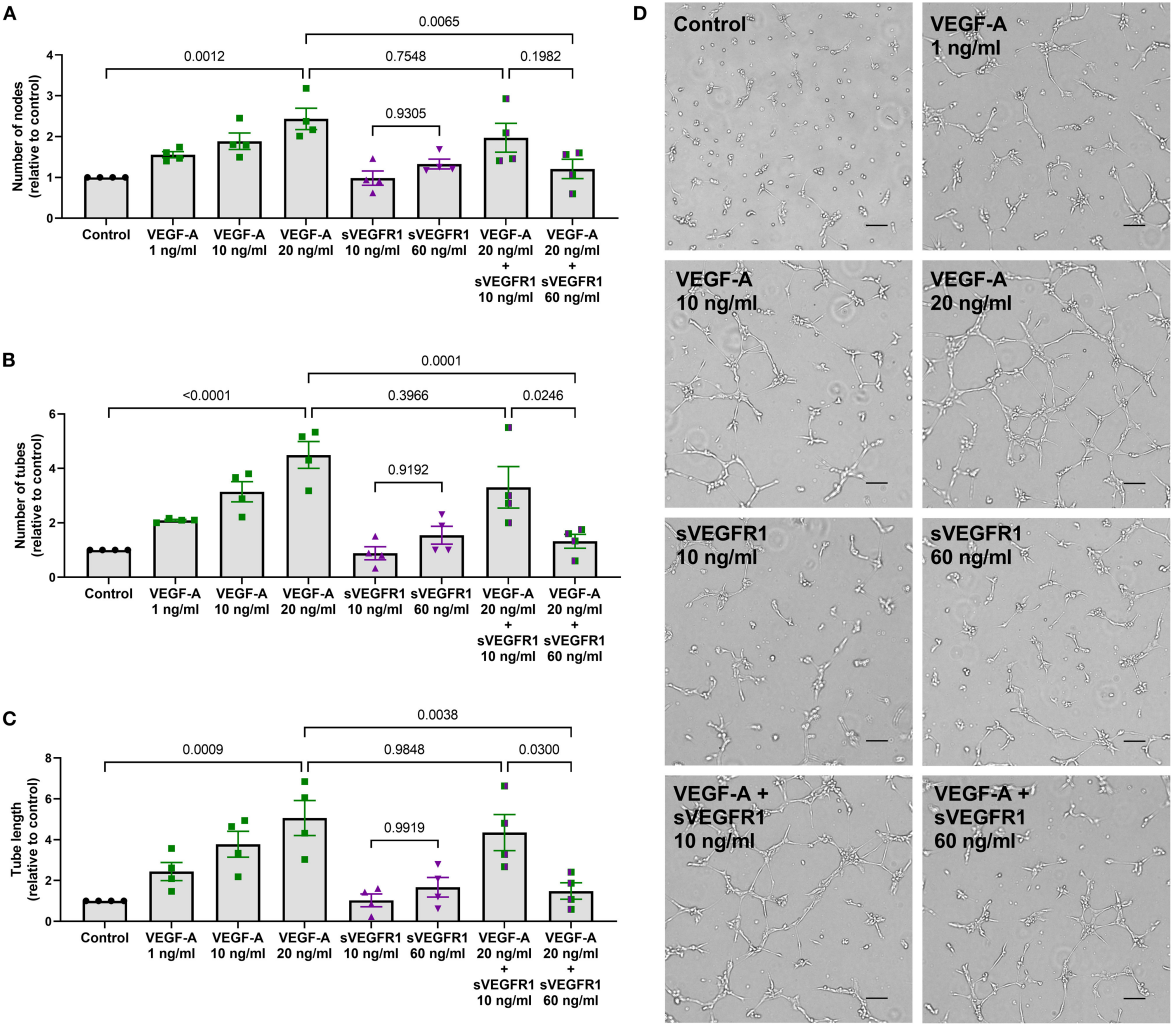
*In vitro* tube formation assay. **(A–C)** Scatter dot plots with bars quantifying the tube formation with various treatments: control, VEGF-A alone (1, 10, or 20 ng/ml), sVEGFR1 alone (10 or 60 ng/ml), or VEGF-A (20 ng/ml) + sVEGFR1 (10 or 60 ng/ml). Tube formation (nodes, tubes, and tube length) increased with increasing VEGF-A concentration. There was no difference in tube formation with the different sVEGFR1 treatments alone. VEGF-A stimulated HPMECs had significantly fewer nodes (*p* = 0.0065), fewer tubes (*p* = 0.0001), and shorter tube length (*p* = 0.0038) after HV-related sVEGFR1 treatment (60 ng/ml). VEGF-A stimulated HPMECs were uninhibited by SVC-related sVEGFR1 treatment (10 ng/ml) (nodes: *p* = 0.7548; tubes: *p* = 0.3966; tube length: *p* = 0.9848) (one-way ANOVA with Tukey multiple comparison test). **(D)** Representative images of tube formation for each treatment. Scale bar indicates 100 μm. Mean ± SEM. HPMECs- human pulmonary microvascular endothelial cells.

After identifying differences in a tip cell population marker, we then wanted to test whether the mean concentrations of sVEGFR1 in HV and SVC serum lead to disparate degrees of angiogenesis *in vitro*. To do this, we performed the tube formation assay, which is an established *in vitro* angiogenesis assay (15). We observed that increasing doses of VEGF-A (1, 10, and 20 ng/ml) increase tube formation *in vitro* by increasing the number of nodes, number of tubes, and tube length (Figures 3A–D). Then, similar to the effect of sVEGFR1 concentration on tip cell selection, we found that our HV-related sVEGFR1 treatment (60 ng/ml) decreased tube formation of VEGF stimulated (20 ng/ml) HPMECS compared to VEGF stimulation alone (nodes: *p* = 0.0065, tubes: *p* = 0.0001, tube length: *p* = 0.0038), whereas SVC-related sVEGFR1 treatment (10 ng/ml) did not decrease tube formation compared to VEGF stimulation alone (nodes: *p* = 0.7548, tubes: *p* = 0.3966, tube length: *p* = 0.9848) (Figures 3A–D). HV-related sVEGFR1 treatment also decreased tube formation of VEGF-stimulated HPMECs compared to SVC-related sVEGFR1 treatment (Figures 3A–D). Specifically, HV-related treatment significantly decreased the number of tubes (HV: 1.32 ± 0.26, SVC: 3.30 ± 0.76, *p* = 0.0246, Figure 3B) and tube length (HV: 1.49 ± 0.41, SVC: 4.35 ± 0.88, *p* = 0.0300, Figure 3C), but non-significantly decreased the number of nodes (HV: 1.21 ± 0.24, SVC: 1.97 ± 0.35, *p* = 0.1982, Figure 3A). Treatment with sVEGFR1 alone (10 or 60 ng/ml) without simultaneous VEGF-A stimulation again showed no difference in tube formation between the different sVEGFR1 concentrations (Figures 3A–D). Altogether, these results show that the quantified sVEGFR1 concentrations in HV and SVC serum differentially influence lung microvascular tube formation *in vitro*. Thus, based on *in vitro* tube formation with lung microvascular endothelial cells, sVEGFR1 concentrations in SVC serum led to uninhibited angiogenesis compared to sVEGFR1 concentrations in HV serum.

## Discussion

Using three independent assays, our data demonstrate that circulating sVEGFR1, also known as soluble fms related receptor tyrosine kinase 1 (sFLT1), is enriched in HV serum compared to SVC serum of children with univentricular and biventricular CHD. sVEGFR1 is known to sequester free VEGF-A to prevent endothelial VEGF signaling. Thus, sVEGFR1 has a possible role as hepatic factor because VEGF-A and VEGFR2 expression are increased in lung biopsies of children with CHD-associated PAVMs (9), and supplemental sVEGFR1 can prevent brain AVMs in a mouse model of AVMs (16). In fact, others have even hypothesized that a circulating VEGF inhibitor may be the unidentified hepatic factor (17). Thus, we propose that sVEGFR1 is a provisional hepatic factor candidate.

Elevated levels of sVEGFR1 are associated with preeclampsia (18, 19), a life-threatening obstetrical condition with systemic hypertension and renal dysfunction. Compared to recent studies, our HV levels of sVEGFR1 (quantitative protein array: 101.26 [90.12, 179.49] ng/ml; ELISA: 62.43 ± 10.97 ng/ml) are similar to peripheral venous levels of sVEGFR1 from mothers with preeclampsia (~80 ng/ml) (20) and are higher than peripheral venous levels in healthy adult controls (~0.1–10 ng/ml) (20, 21). These comparisons suggest that elevated levels of sVEGFR1 in HV serum may have a functional role in the downstream pulmonary capillary bed.

In addition to absolute differences in sVEGFR1 concentration between HV and SVC serum, our data demonstrate that the observed HV and SVC concentrations of sVEGFR1 differentially impact primary human pulmonary microvascular endothelial cells *in vitro*. Using short-term *in vitro* read-outs, we observed that VEGF-stimulated pulmonary microvascular endothelial cells were uninhibited by SVC-related sVEGFR1 concentration. In contrast, HV-related sVEGFR1 concentration decreased tip cell selection (Figure 2) and tube formation (Figure 3). It is unknown if these differences are directly relevant to CHD-associated PAVMs, though these processes are consistent with previously published data. Using a rat model of superior cavopulmonary circulation (classic Glenn palliation), Tipps et al. (22) reported increased expression of the tip cell marker *ESM1* in CHD-associated PAVMs, which is similar to our data that the lower sVEGFR1 concentration found in SVC serum leads to increased CD34 population *in vitro*. Thus, prolonged exposure of the pulmonary vascular bed to exclusive SVC blood flow (i.e., superior cavopulmonary circulation) may lead to uninhibited tip cell selection. As a potential mechanism, we speculate that when liver-derived sVEGFR1 cannot perfuse the pulmonary microvasculature, the normal angiogenic balance in the lung microenvironment is lost. This angiogenic imbalance predisposes to pathologic angiogenesis with abnormal vascular connections and AVMs. We do not know if systemic levels of VEGF-A are part of this process, as opposed to local tissue VEGF-A levels.

To our knowledge, this study includes the broadest proteomic comparison (>440 proteins) of HV and SVC serum from children with univentricular and biventricular CHD. The unbiased quantitative serum protein array in this study permits data-driven consideration of alternative hepatic factor candidates, as well as analysis of SVC serum. For example, our quantitative array preliminarily indicates that SVC serum may be enriched in pro-inflammatory cytokines (IFNγ, TNFB), angiogenic factors (BDNF), and lymphatic growth factors (VEGF-C). AVM pathogenesis in patients with hereditary predisposition to AVMs (i.e., hereditary hemorrhagic telangiectasia; HHT) appears to require a two-hit process for AVM development (23). Proposed second hits include mechanical trauma, inflammation, and angiogenic cues. It is unknown if two-hits are necessary for development of CHD-associated PAVMs (loss of hepatic factor, AND hypoxia, non-pulsatile pulmonary blood flow, inflammation, etc.). Given that our proteomic array preliminarily identified that several pro-inflammatory cytokines are enriched in SVC blood, the contribution of SVC blood to PAVM pathophysiology may be under-recognized. In fact, the presence of pro-inflammatory cytokines in SVC serum may help explain the previous findings that SVC blood increases pulmonary microvascular endothelial cell apoptosis *in vitro*, but it is unclear whether this addresses the previous surprising finding that HV serum increases tube formation *in vitro* compared to SVC serum (5). Regardless, in this study, our quantitative protein array demonstrates that HV and SVC serum samples contain a mix of proteins with diverse functions.

Both our semi-quantitative immunoblot and quantitative protein array also confirm the recent finding (7) that BMP9 is not elevated in HV blood. These data collectively indicate that CHD-associated PAVMs appear to develop independent of the upstream signaling pathway involved in HHT-associated PAVMs (BMP9-ALK1 signaling). Importantly, we cannot rule-out overlap in PAVM pathogenesis in the downstream signaling. In fact, HHT animal models indicate that VEGF inhibition can partially reverse AVM pathology (24–26), and bevacizumab, a VEGF-A monoclonal neutralizing antibody, provides therapeutic benefit for patients with HHT (27, 28). Altogether, these data further support that sVEGFR1 may have a role in PAVM pathophysiology given that sVEGFR1 sequesters VEGF-A to decrease VEGF-A bioavailability and signaling.

Two recent studies have also reported that Angiopoietin-2 is elevated in patients with palliated univentricular CHD compared to patients with biventricular CHD (6, 29). Angiopoietin-2 is associated with vascular remodeling, but these studies found no difference in ANG-2 levels in HV blood compared to paired SVC blood. Shirali et al. (6) even concluded that ANG2 elevation is likely unrelated to PAVM formation. Similarly, our quantitative array found similar levels of ANG2 in HV and SVC serum (HV: 5265.91 [2189.98, 7908.51] pg/ml, SVC: 3981.83 [2511.55, 7467.13] pg/ml). On the other hand, ANG1 is associated with promoting vascular homeostasis. Bartoli et al. (29) reported decreased levels of ANG1 in SVC plasma compared to HV plasma and compared to healthy controls. Our quantitative array showed contradictory results with increased ANG1 levels in SVC serum compared to HV serum (HV: 1774.20 [665.51, 2251.92] pg/ml, SVC: 5755.66 [3237.47, 7933.60] pg/ml; Figure 1C, Supplementary Table 1), although these data are from a small sample (*n* = 7). Finally, an important note is that sVEGFR1 was not quantified in either of these recent studies.

Our preliminary findings are fundamentally limited by our overall small, heterogenous cohort, which may predispose to sample bias. We included patients with univentricular and biventricular anatomy under the premise that hepatic factor is present in all patients regardless of ventricular anatomy; however, patients with biventricular anatomy may have differing physiology impacting pulmonary vascular status and serum protein levels, which may ultimately skew results. Additionally, blood samples were collected in the cardiac catheterization lab to identify constituents of specific venous blood sources, but multiple procedure-related factors may influence blood composition. For example, general anesthesia, pre-procedure fasting, or other confounding factors may directly or indirectly alter venous blood compared to normal physiologic conditions. Validation of our findings with an independent, larger cohort with longitudinal sample collection and longer-term follow-up is needed. Also, because traditional *in vitro* assays are relatively insensitive, we used a standard *in vitro* concentration of VEGF-A (20 ng/ml) to elicit a quantifiable and reproducible endothelial cell response. This concentration of VEGF-A was much greater than our quantified circulating level of VEGF-A (~40 pg/ml; Supplementary Table 1), which limits the physiologic relevance of our endothelial cell read-outs. Finally, our analyses of HV and SVC serum are limited to protein arrays because hepatic factor has previously been hypothesized to be a singular circulating protein; however, we acknowledge that multiple factors (including non-protein factors) may interact to fulfill the function of the unidentified hepatic factor. The potential interaction of multiple factors may help explain the apparent discrepant findings from our previous study that HV serum increases EC tube formation *in vitro*, whereas in this study, we report that sVEGFR1 is enriched in HV serum and decreases EC tube formation *in vitro*.

In conclusion, our data indicate that sVEGFR1 is enriched in HV serum compared to SVC serum. The observed differences in sVEGFR1 serum concentrations also lead to pulmonary microvascular endothelial phenotypic differences *in vitro*. Further investigation is needed to experimentally determine whether sVEGFR1 has a direct role in CHD-associated PAVMs.

## Data Availability Statement

The raw data supporting the conclusions of this article will be made available by the authors, without undue reservation.

## Ethics Statement

The studies involving human participants were reviewed and approved by Children's Hospital of Wisconsin Institutional Review Board. Written informed consent to participate in this study was provided by the participants' legal guardian/next of kin.

## Author Contributions

AS: Participated in study design, performed and conceptualized experiments, performed statistical analysis, interpreted data, wrote and edited manuscript. AG: Participated in study design, performed and conceptualized experiments. AP: Participated in study design, performed statistical analysis, helped write and edit parts of the manuscript. TG and SF: Participated in study design, helped edit parts of the manuscript. GK: Participated in study design, provided intellectual input, helped edit parts of the manuscript. RR: Participated in study design, provided intellectual input, helped conceptualize experiments, helped interpret data, helped edit parts of the manuscript. All authors contributed to the article and approved the submitted version.

## Conflict of Interest

The authors declare that the research was conducted in the absence of any commercial or financial relationships that could be construed as a potential conflict of interest.

## Supplementary Material

Supplementary Figure 1 Overall and sub-group comparisons of sVEGFR1 levels quantified using sandwich ELISA. Paired serum levels (ng/ml) of sVEGFR1 in the overall ELISA cohort (*n* = 24) and sub-groups [PAVMs (*n* = 9), no PAVMs (*n* = 15), univentricular congenital heart disease (*n* = 11), and biventricular congenital heart disease (*n* = 14)]. Paired *t*-tests.

Supplementary Table 1 Quantitative serum protein array.

Supplementary Table 2 Effects of patient characteristics on sVEGFR1 serum concentration.
