# Supplementary figures and images for: sVEGFR1 Is Enriched in Hepatic Vein Blood—Evidence for a Provisional Hepatic Factor Candidate?
Source: Front Pediatr. 2021 Jun 14;9:679572. doi: 10.3389/fped.2021.679572 (PMC8236596; doi:10.3389/fped.2021.679572)

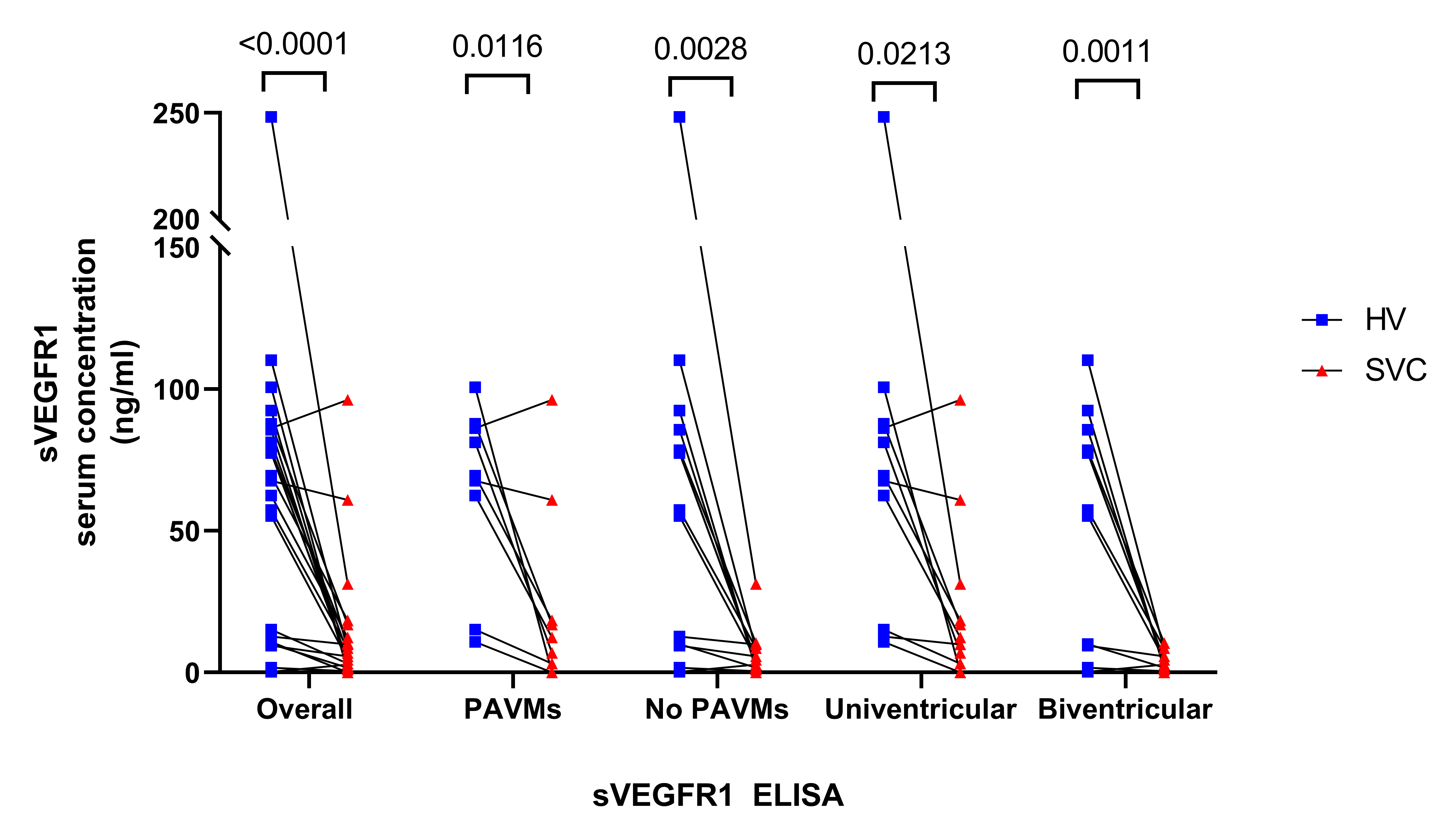

Supplement: Supplementary Figure 1 — Overall and sub-group comparisons of sVEGFR1 levels quantified using sandwich ELISA. Paired serum levels (ng/ml) of sVEGFR1 in the overall ELISA cohort (n = 24) and sub-groups [PAVMs (n = 9), no PAVMs (n = 15), univentricular congenital heart disease (n = 11), and biventricular congenital heart disease (n = 14)]. Paired t-tests. [file Image_1.TIF]
