# Supplementary material for: sVEGFR1 Is Enriched in Hepatic Vein Blood—Evidence for a Provisional Hepatic Factor Candidate?
Source: Front Pediatr. 2021 Jun 14;9:679572. doi: 10.3389/fped.2021.679572 (PMC8236596; doi:10.3389/fped.2021.679572)
Supplement: Supplementary Table 1 — Quantitative serum protein array. [file Table_1.docx]

| Supplemental Table 1: Quantitative serum protein array | | | | | | |
| --- | --- | --- | --- | --- | --- | --- |
| Protein Target | **UniProt ID** | **HV** | **SVC** | **Average Log2 Fold Change** | **Raw p-value** | **FDR** |
| ENA-78 | P42830 | 1429.44 [468.54, 1773.44] | 1912.27 [1535.49, 2523.15] | -0.86 | 0.0156 | 0.1901 |
| Fas | P25445 | 309.88 [201.58, 494.98] | 352.99 [207.25, 536.77] | -0.2 | 0.0156 | 0.1901 |
| IFNγ | P01579 | 45.16 [29.81, 118.55] | 317.36 [285.51, 362.32] | -2.03 | 0.0156 | 0.1901 |
| IGFBP-3 | P17936 | 33310.74 [29483.07, 58049.57] | 28279.10 [25399.02, 43417.98] | 0.38 | 0.0156 | 0.1901 |
| IL-13 | P35225 | 3.42 [2.55, 9.83] | 22.94 [14.95, 32.46] | -1.55 | 0.0156 | 0.1901 |
| IL-17 | Q16552 | 5.34 [4.44, 10.74] | 31.11 [19.27, 38.49] | -1.83 | 0.0156 | 0.1901 |
| IL-17B | Q9UHF5 | 431.69 [76.73, 776.28] | 1830.14 [783.14, 3220.60] | -2.75 | 0.0156 | 0.1901 |
| IL-17R | Q96F46 | 186.20 [61.10, 540.98] | 20.44 [0.00, 492.12] | 2.93 | 0.0156 | 0.1901 |
| IL-1b | P01584 | 3.87 [2.52, 58.22] | 10.43 [3.61, 78.98] | -0.39 | 0.0156 | 0.1901 |
| IL-1ra | P18510 | 8.73 [7.83, 31.41] | 21.12 [17.34, 59.78] | -1.11 | 0.0156 | 0.1901 |
| IL-2 | P60568 | 195.02 [93.50, 356.36] | 1032.88 [692.66, 1094.82] | -1.85 | 0.0156 | 0.1901 |
| IL-31 | Q6EBC2 | 151.76 [22.61, 4316.26] | 232.15 [22.79, 4490.32] | -0.43 | 0.0156 | 0.1901 |
| IL-5 | P05113 | 42.82 [28.73, 105.15] | 315.47 [207.78, 379.99] | -1.94 | 0.0156 | 0.1901 |
| IL-7 | P13232 | 24.29 [19.03, 56.92] | 158.15 [120.99, 185.69] | -1.72 | 0.0156 | 0.1901 |
| LIGHT | O43557 | 64.86 [13.58, 318.24] | 210.98 [71.79, 368.66] | -1.23 | 0.0156 | 0.1901 |
| MCP-2 | P80075 | 85.46 [60.20, 278.68] | 226.89 [127.19, 464.14] | -1.09 | 0.0156 | 0.1901 |
| MCSF-R | P07333 | 15665.93 [13008.12, 16251.81] | 14017.54 [12866.60, 15111.76] | 0.11 | 0.0156 | 0.1901 |
| MICA | Q29983 | 681.21 [468.30, 1311.47] | 739.92 [580.19, 1599.27] | -0.32 | 0.0156 | 0.1901 |
| MIP-1b | P13236 | 14.28 [12.61, 27.20] | 23.56 [14.69, 35.66] | -0.73 | 0.0156 | 0.1901 |
| MIP-1d | Q16663 | 1189.94 [1077.73, 1628.39] | 1433.02 [1153.01, 1716.87] | -0.09 | 0.0156 | 0.1901 |
| Midkine | P21741 | 5552.38 [4887.54, 14920.63] | 3433.03 [2000.58, 13818.77] | 0.62 | 0.0156 | 0.1901 |
| NCAM-1 | P13591 | 158012.44 [151481.78, 180682.84] | 196218.60 [159829.98, 205415.58] | -0.21 | 0.0156 | 0.1901 |
| PDGF-AA | P04085 | 1053.79 [539.25, 1595.01] | 1757.34 [858.32, 2058.33] | -0.87 | 0.0156 | 0.1901 |
| PF4 | P02776 | 63477.26 [59035.65, 67457.22] | 25153.11 [21096.09, 62832.57] | 1.04 | 0.0156 | 0.1901 |
| sVEGFR1 | P17948 | 101255.25 [90124.17, 179491.16] | 13206.39 [8035.04, 92525.08] | 2.09 | 0.0156 | 0.1901 |
| TARC | Q92583 | 166.86 [72.82, 257.12] | 406.43 [230.17, 769.00] | -1.29 | 0.0156 | 0.1901 |
| TIMP-2 | P16035 | 22377.87 [19351.49, 25004.99] | 18539.66 [17531.45, 20907.44] | 0.22 | 0.0156 | 0.1901 |
| TNFB | P01374 | 243.37 [161.33, 721.44] | 1880.97 [1317.56, 2153.89] | -2.07 | 0.0156 | 0.1901 |
| TPO | P40225 | 1532.84 [561.01, 2111.59] | 2472.67 [990.21, 3085.63] | -1.41 | 0.0156 | 0.1901 |
| TSLP | Q969D9 | 55.66 [2.09, 1509.27] | 68.12 [18.29, 1909.13] | -1.04 | 0.0156 | 0.1901 |
| TSP-1 | P07996 | 23185.35 [22452.52, 24109.71] | 24320.51 [22854.69, 28465.36] | -0.14 | 0.0156 | 0.1901 |
| TSP-5 | P49747 | 1817.78 [1675.18, 2555.19] | 1579.54 [996.91, 2018.88] | 0.42 | 0.0156 | 0.1901 |
| ANG-1 | Q15389 | 1774.20 [665.51, 2251.92] | 5755.66 [3237.47, 7933.60] | -1.68 | 0.0312 | 0.1901 |
| Albumin | P02768 | 739.88 [645.16, 883.75] | 841.63 [804.33, 876.15] | -0.17 | 0.0312 | 0.1901 |
| BDNF | P23560 | 45.71 [18.60, 143.03] | 469.35 [146.50, 672.19] | -2.52 | 0.0312 | 0.1901 |
| BLC | O43927 | 61.17 [22.98, 123.66] | 59.97 [31.71, 125.56] | -0.4 | 0.0312 | 0.1901 |
| BMP-2 | P12643 | 520.96 [313.58, 1381.70] | 1591.20 [921.43, 2744.93] | -1.01 | 0.0312 | 0.1901 |
| CKB-8 | P55773-2 | 7780.70 [2532.46, 14242.51] | 65.21 [0.00, 9581.48] | 4.43 | 0.0312 | 0.1901 |
| DR6 | O75509 | 7697.52 [4257.50, 10271.43] | 8081.96 [4365.35, 10396.13] | -0.08 | 0.0312 | 0.1901 |
| EGF | P01133 | 1.87 [0.59, 2.13] | 2.61 [1.67, 5.52] | -0.63 | 0.0312 | 0.1901 |
| E-selectin | P16581 | 75050.48 [42029.89, 95970.93] | 105203.22 [87524.05, 127639.59] | -0.58 | 0.0312 | 0.1901 |
| Eotaxin | P51671 | 25.91 [17.44, 45.40] | 33.60 [32.32, 71.41] | -0.65 | 0.0312 | 0.1901 |
| GM-CSF | P04141 | 42.26 [24.29, 123.80] | 273.59 [212.95, 330.05] | -1.83 | 0.0312 | 0.1901 |
| HAI-2 | O43291 | 87.03 [0.00, 264.19] | 219.79 [79.63, 559.62] | -2.14 | 0.0312 | 0.1901 |
| IL-10 | P22301 | 14.25 [7.27, 20.45] | 34.77 [32.26, 40.41] | -1.08 | 0.0312 | 0.1901 |
| IL-11 | P20809 | 9.64 [0.26, 20.51] | 45.85 [32.48, 80.16] | -2.16 | 0.0312 | 0.1901 |
| IL-12p70 | P29459 | 0.62 [0.24, 2.77] | 1.25 [1.13, 3.30] | -0.48 | 0.0312 | 0.1901 |
| IL-15 | P40933 | 17.52 [10.43, 332.93] | 41.52 [27.94, 359.19] | -0.5 | 0.0312 | 0.1901 |
| IL-17E | Q9H293 | 2456.54 [1258.71, 6226.87] | 2147.14 [950.44, 4751.29] | 0.42 | 0.0312 | 0.1901 |
| IL-6 | P05231 | 37.98 [12.59, 154.99] | 125.17 [89.08, 243.30] | -1.5 | 0.0312 | 0.1901 |
| IL-8 | P10145 | 13.91 [3.77, 64.90] | 44.33 [24.06, 80.04] | -0.9 | 0.0312 | 0.1901 |
| I-309 | P22362 | 9.19 [4.70, 64.00] | 27.58 [11.56, 83.86] | -1.12 | 0.0312 | 0.1901 |
| LAP-TGFβ1 | P01137.2 | 1461.59 [1052.59, 2238.80] | 3130.90 [2121.92, 3442.76] | -0.73 | 0.0312 | 0.1901 |
| LIF | P15018 | 22.04 [4.66, 185.11] | 58.75 [26.67, 253.19] | -0.97 | 0.0312 | 0.1901 |
| Lipocalin-2 | P80188 | 1781.08 [1568.94, 2227.03] | 1960.12 [1919.33, 2134.38] | -0.17 | 0.0312 | 0.1901 |
| MCP-1 | P13500 | 521.36 [361.15, 727.63] | 1083.82 [930.22, 1525.18] | -0.93 | 0.0312 | 0.1901 |
| MCP-3 | P80098 | 0.00 [0.00, 28.06] | 13.58 [4.63, 46.87] | -2.08 | 0.0312 | 0.1901 |
| MCP-4 | Q99616 | 25.11 [13.52, 36.17] | 60.19 [20.80, 76.41] | -0.83 | 0.0312 | 0.1901 |
| MIG | Q07325 | 2177.64 [1305.78, 3142.71] | 989.73 [152.63, 2371.73] | 0.99 | 0.0312 | 0.1901 |
| MMP-1 | P03956 | 2457.82 [813.98, 4686.45] | 4544.20 [3053.48, 7897.21] | -0.89 | 0.0312 | 0.1901 |
| Nectin-4 | Q96NY8 | 119.77 [3.31, 752.36] | 0.00 [0.00, 631.49] | 2.96 | 0.0312 | 0.1901 |
| OPN | P10451 | 3724.22 [3216.98, 4254.69] | 2058.31 [1725.45, 3548.22] | 0.67 | 0.0312 | 0.1901 |
| PDGF-AB | P04085 | 3901.61 [1965.06, 7749.07] | 7768.02 [6154.62, 9464.81] | -0.87 | 0.0312 | 0.1901 |
| PDGF-BB | P01127 | 1160.17 [940.30, 2029.78] | 3004.30 [2727.42, 3468.73] | -0.94 | 0.0312 | 0.1901 |
| Periostin | Q15063 | 16772.25 [11069.79, 36264.64] | 19315.02 [11112.11, 45265.01] | -0.26 | 0.0312 | 0.1901 |
| SDF-1a | P48061 | 47.28 [29.57, 95.16] | 39.61 [13.79, 68.81] | 0.41 | 0.0312 | 0.1901 |
| SDF-1b | P48061 | 301.75 [58.06, 435.37] | 88.11 [59.94, 375.30] | 0.66 | 0.0312 | 0.1901 |
| Syndecan-4 | P31431 | 32.88 [21.95, 108.52] | 63.82 [60.98, 155.43] | -0.61 | 0.0312 | 0.1901 |
| TNFα | P01375 | 115.08 [66.50, 180.50] | 546.73 [419.80, 619.66] | -1.65 | 0.0312 | 0.1901 |
| ULBP-2 | Q9BZM5 | 195.11 [94.76, 737.63] | 115.95 [41.17, 677.97] | 0.94 | 0.0312 | 0.1901 |
| VEGF-A | P15692 | 43.07 [6.67, 68.89] | 40.21 [35.59, 115.26] | -1.28 | 0.0312 | 0.1901 |
| VEGF-C | P49767 | 9.85 [0.00, 42.19] | 76.66 [29.96, 90.14] | -2.42 | 0.0312 | 0.1901 |

Serum protein concentrations (pg/ml) in hepatic vein (HV) and superior vena cava (SVC) serum; median [IQR]. Raw p-values calculated using Wilcoxon signed ranked test. FDR: false discovery rate. Proteins listed are those with an FDR < 0.2. Samples are listed in descending order based on raw p-values.
