# Supplementary material for: sVEGFR1 Is Enriched in Hepatic Vein Blood—Evidence for a Provisional Hepatic Factor Candidate?
Source: Front Pediatr. 2021 Jun 14;9:679572. doi: 10.3389/fped.2021.679572 (PMC8236596; doi:10.3389/fped.2021.679572)
Supplement: Supplementary Table 2 — Effects of patient characteristics on sVEGFR1 serum concentration. [file Table_2.docx]

Supplemental Table 2: Effects of patient characteristics on sVEGFR1 serum concentration

| Effect | P value* | P value** |
| --- | --- | --- |
| Group (HV vs SVC) | <0.0001 | <0.0001 |
| Age | 0.30 | 0.17 |
| Gender | 0.78 | 0.51 |
| Ventricular anatomy | 0.10 | 0.39 |
| SpO_2_ | 0.081 | 0.79 |

*Group and an individual patient’s characteristics variable in the model

**Group and all patients’ characteristics variables in the model

HV= hepatic vein, SpO_2_= Peripheral oxygen saturation, SVC= superior vena cava.
